# Predictors of dropout from care among HIV-infected patients initiating antiretroviral therapy at a public sector HIV treatment clinic in sub-Saharan Africa

**DOI:** 10.1186/s12879-016-1392-7

**Published:** 2016-02-01

**Authors:** Stephen B. Asiimwe, Michael Kanyesigye, Bosco Bwana, Samson Okello, Winnie Muyindike

**Affiliations:** 1 Department of Medicine, Mbarara Regional Referral Hospital, P.O Box 40 Mbarara, Uganda; 2 Department of Epidemiology and Biostatistics, University of California San Francisco, California, USA; 3 Department of Medicine, Mbarara University of Science and Technology, Mbarara, Uganda

**Keywords:** HIV treatment, Dropout, Loss to follow-up, Antiretroviral therapy, Sub-Saharan Africa, Predictors

## Abstract

**Background:**

In sub-Saharan Africa (SSA), antiretroviral therapy (ART) can prolong life for HIV-infected patients. However, patients initiating ART, especially in routine treatment programs, commonly dropout from care either due to death or loss to follow-up.

**Methods:**

In a cohort of HIV-infected patients initiating ART at a public sector clinic in Uganda, we assessed predictors of dropout from care (a composite outcome combining death and loss to follow-up). From a large set of socio-demographic, clinical, and laboratory variables routinely collected at ART initiation, we selected those predicting dropout at P <0.1 in unadjusted analyses for inclusion into a multivariable proportional hazards regression model. We then used a stepwise backward selection procedure to identify variables which independently predicted dropout at P <0.05.

**Results:**

Data from 5,057 patients were analyzed. The median age was 33 years (IQR 28 to 40) and 27.4 % had CD4+ T-cell counts <100 cells/μL at ART initiation. The median duration of follow-up was 24 months (IQR = 14 to 42, maximum follow-up = 64 months). Overall dropout was 26.9 % (established cumulative mortality = 2.3 %, loss to follow-up = 24.6 %), 5.6 % were transferred to other service providers, and 67.5 % were retained in care. A diagnosis of Kaposi’s sarcoma (hazard ratio (HR) = 3.3, 95 % CI 2.5 to 4.5); HIV-associated dementia (HR = 2.6, 95 % CI 1.5 to 4.6); history of cryptococcosis (HR = 2.2, 95 % CI 1.4 to 3.3); and reduced hemoglobin concentration (<11 g/dl versus ≥13.8 g/dl (HR = 1.9, 95 % CI 1.6 to 2.2) were strong predictors of dropout. Other independent predictors of dropout were: year of ART initiation; weight loss ≥10 %; reduced total lymphocyte count; chronic diarrhea; male sex; young age (≤28 years); and marital status.

**Conclusions:**

Among HIV-infected patients initiating ART at a public sector clinic in SSA, biological factors that usually predict death were especially predictive of dropout. As most of the dropouts were lost to follow-up, this observation suggests that many losses to follow-up may have died. Future studies are needed to identify appropriate interventions that may improve both individual-level patient outcomes and outcome ascertainment among HIV-infected ART initiators in this setting.

## Background

Sub-Saharan Africa (SSA) remains the epicenter of the HIV pandemic. In recent data, the prevalence of HIV infection averaged around 18 % to 26 % in Southern Africa and 5.1 % to 7.4 % in East Africa [1]. In Uganda, where HIV prevalence in adults was initially as high as 30 % in 1990 (based on data from antenatal care clinics) [2], aggressive prevention campaigns achieved an initial dramatic decline in the prevalence to about 4.1 % at the end of 2003 [3]. However, recently, the HIV prevalence has increased again to about 7.4 % in 2013 [2]. Although HIV incidence appears to be declining in general in SSA [4, 5], the number of new infections remains high [6].

For the past decade, antiretroviral therapy (ART) has been rolled out in the region [7]. ART can prolong life for HIV-infected patients [8], and is also a major opportunity for HIV prevention since successful treatment of those already infected can prevent continued HIV transmission [9]. However, significant challenges remain in the implementation and evaluation of HIV treatment services in SSA. In particular, among patients initiating ART, dropout from treatment is common (as high as 30–50 % at 1 year in some programs) [10, 11]. The majority of dropouts are due to loss to follow-up [12]. High rates of loss to follow-up are an important threat to the success of HIV treatment programs; patients that are lost can interrupt their treatment, resulting in further disease progression, continued HIV transmission, and death [13]. Also, high rates of loss to follow-up complicate program evaluation by biasing mortality estimates [14, 15]. Interventions are thus needed to improve not only individual-level patient outcomes but also outcome ascertainment for HIV infected patients initiating ART in the region.

Dropout from care is especially common for those receiving ART from routine treatment programs [11]. As these programs usually collect a relatively large amount of sociodemographic, clinical, and laboratory data from patients at ART initiation, we hypothesized that such data could be used to pre-emptively identify patients at high risk of dropout, who in turn might benefit from interventions to promote retention. We therefore aimed to evaluate predictors of dropout from care among HIV-infected patients initiating ART at a public-sector clinic in Uganda.

## Methods

### Study setting, design, and population

We performed a cohort analysis of data from the electronic database of the Immune Suppression Syndrome (ISS) clinic in Mbarara, Uganda. Dropout from care among patients on ART is common at this clinic [16, 17]. HIV treatment services, which are free to patients, are paid for by the President’s Emergency Fund for AIDS Relief through the US Centers for Disease Control, the Uganda Ministry of Health, and the Makerere University-Mbarara University Joint AIDS Program. We included in this analysis, the data of patients that were 16 years and older, who initiated ART between January 2008 and December 2011. We chose this period so that we would have adequate baseline and follow-up data available in the electronic database.

#### Ethics statement

We used de-identified data that had already been collected for the patients’ clinical care. As it would have been either inconveniencing to the patients or difficult to reach them, we did not seek individual-level consent from patients to use these data. Instead, we planned to access only de-identified data and sought a waiver on the requirement for individual-level consent from the local Institutional Review Board (IRB). This waiver was granted pre-analysis by the Mbarara University of Science and Technology Institutional Review Board (MUST-IRB). The data management committee of the ISS Clinic also approved the study.

### Data collection procedures

Since 2007, clinic data routinely collected by nurses, counselors, physicians, and pharmacy staff on paper have been subsequently entered into an electronic database maintained in the Open Medical Records System [18]. For this analysis, the clinic’s data manager extracted de-identified patient data on our behalf.

### Predictor measurements

We investigated socio-demographic, clinical, and laboratory variables that are routinely measured at ART initiation as predictors of dropout. Socio-demographic variables included age, sex, education level, time spent travelling to the clinic, and marital status. Clinical variables included histories/diagnoses of opportunistic infections (OIs) and other comorbidities at ART initiation. We assessed only those disorders that are commonly diagnosed in this setting, and which we expected to be diagnosed with relatively good specificity. These disorders included: tuberculosis (TB), Kaposi’s sarcoma (KS), cryptococcosis, esophagitis, oral candidiasis, chronic diarrhea, HIV-associated dementia, and weight loss ≥10 %.

At this clinic, TB is diagnosed using laboratory (Ziehl-Neelsen sputum smears), clinical (presence of chronic productive cough, weight loss, night sweats, and abnormal chest examination findings), and radiological assessments (abnormal findings on chest radiographs suggestive of TB, e.g., a "miliary" pattern of small nodules in the lung parenchyma). TB cases are defined according to standard criteria [19]; diagnoses for patients that are sputum-smear negative require consensus from at least 2 clinicians. KS diagnoses are based on clinical examination for oral lesions and skin biopsy for skin lesions. Skin biopsies are followed by off-site histopathological examination [20]. Diagnoses for cryptococcosis are based on serum cryptococcal antigen (CRAG), cerebrospinal fluid (CSF) CRAG, and CSF India ink tests [21] (although these tests are done only for patients with symptoms suggestive of chronic meningeal irritation). Diagnoses of esophagitis, HIV-associated dementia, and weight loss are based on clinical reports.

Laboratory measurements included complete blood count (CBC) characteristics (hemoglobin concentration, neutrophil counts, total lymphocyte counts (TLC), eosinophil counts, and platelet counts) and CD4+ T cell counts (Coulter AC five-part differential, and Coulter Epics Cytometer, Beckman Coulter, Brea, California) measured at ART initiation (±3 months). We did not assess other laboratory parameters, such as, the plasma HIV RNA levels and liver or kidney function tests, because these measurements were rarely performed on patients.

#### Antiretroviral therapy regimens

Data on the patients’ initial ART regimens were also obtained. At this clinic an initial ART regimen contains at least 3 antiretroviral drugs; a backbone drug, usually a nucleoside- or nucleotide-analog reverse transcriptase inhibitor (NRTI), which can be zidovudine (AZT) (the most common), tenofovir (TDF), or stavudine (D4T); a second drug also often a NRTI, which can be either lamivudine (3TC) or emtricitabine (FTC); and a third drug, usually either a non-nucleoside reverse transcriptase inhibitor (NNRTI) in the form of nevirapine (NVP) or Efavirenz (EFV), or a protease inhibitor (PI) (although the PIs are only very rarely used in initial regimens). For example, a common initial regimen would be a combination of AZT, 3TC, and NVP. We obtained data on the specific regimen that each patient received. However, as previous studies suggest that outcomes may vary according to the backbone drug received (e.g., AZT versus TDF) [22], we assessed whether the initial ART regimen may have predicted outcomes by backbone drug received. Consequently, we divided the sample into: those initially receiving AZT-based regimens (alongside either 3TC or FTC and either EFV or NVP); those initially receiving TDF-based regimens (with similar options for the 2nd and 3rd drugs); and those initially receiving D4T-based regimens (this backbone is recently only rarely used, but also has similar options as the others for the 2nd and 3rd drugs).

#### Follow-up and outcomes

Time zero was the date of ART initiation and patients were followed until death, transfer-out to another service provider, loss to follow-up, or administrative censoring on 31 December 2012. The primary outcome was dropout from treatment, a composite of death and loss to follow-up (due to high rates of loss to follow-up in this setting, assessing either outcome alone may potentially lead to biased conclusions; hence we decided to assess both outcomes together as overall “dropout from care”). Participants were considered to be lost to follow-up if their last clinic visit was more than 180 days earlier than the date of administrative censoring in those not transferred out and not known to have died. The 180-day period is based on previous studies defining loss to follow-up in this way [16, 23]. Death was determined through reports by clinic staff, relatives, peers, or friends, since Uganda does not have a national death registry. Clinic staff usually perform limited tracking of patients who dropout from the clinic by inquiring from relatives, peers, and friends, who usually would know if a patient died. Also, patients are at times transferred to other service providers; clinic staff usually document this transfer on the patient’s record (with the information later being captured by the electronic record). In this analysis, such patients were censored at the time of transfer.

For purposes of this analysis, we assumed that death and loss to follow-up were the bad outcomes (hence these were combined), while transfer to another service provider or being retained in care were the good outcomes (these were also combined); our binary outcome of dropout from care thus compares patients who either died or were lost to follow-up to those who were either retained at this clinic or were transferred officially to another provider.

## Analysis

We first performed descriptive analyses on the full sample to assess the degree of missing data and to describe outcomes. We then performed multiple imputations of missing data (as described below) before using proportional hazards regression to assess predictors of dropout.

In unadjusted analyses, we assessed all available sociodemographic, clinical, and laboratory variables. CBC parameters and age were modelled in quartiles; CD4+ T cell count was categorized in clinically-relevant fashion: <100, 100–199, 200–349, and ≥350 cells/μL.

We set up the analysis so that, for each variable, the reference category was that, which we hypothesized pre-analysis to have the lowest risk of dropout. For example, based on some previous studies [24, 25], we hypothesized that older patients on ART are less likely to dropout than younger patients, the married are less likely to dropout than the single, those with higher hemoglobin or higher CD4+ T cell concentrations are less likely to dropout than those with lower concentrations, etc. Consequently, these “normal values” categories were used as the reference per variable. In similar fashion, for variables such as the total lymphocyte counts or the platelet counts, our reference groups were the middle-of-the-range values; we suspected that patients with either reduced or elevated levels of these cell types (i.e., patients with “abnormal” values) would have higher risk of dropout. We then used proportional hazards regression to calculate the hazard ratio (HR) of dropout per variable comparing patients in the other categories to those in the reference category and assessed associated *P* values. The HR in this case is interpretable as a ratio of the instantaneous risk of dropout among patients in a given category versus those in the reference category.

To determine independent predictors of dropout, we included variables that had P values <0.1 in unadjusted analyses in a multivariable proportional hazards regression model predicting dropout. Using a stepwise backward selection procedure, we removed variables from the model until only those that were statistically significantly associated with dropout at P <0.05 remained. As previous studies among HIV infected patients in this setting suggest that calendar time may be associated with death, loss to follow-up, and the quality of data collected from patients [26, 27], we also included the year of ART initiation in the adjusted analysis.

In addition to the main analysis above, which evaluated the patients’ individual-level characteristics as predictors of dropout, we evaluated whether initial ART regimens (based on the backbone drug received) predicted risk of dropout. As some previous studies suggest that TDF- or D4T-based regimens may be more associated with negative outcomes than AZT-based regimens [22, 28], we compared risk of dropout in patients receiving AZT-based regimens (as the reference) to dropout risk in those receiving either TDF-based or D4T-based regimens. In this analysis, we suspected that, at a minimum, sex, age, and year of ART initiation, would be confounders of associations between initial regimen and dropout. Additionally, we suspected that clinicians might be less inclined to give AZT to patients that present with low hemoglobin concentrations, and in one previous study, patients with more advanced HIV disease stage at ART initiation, were more likely to be treated with TDF-based regimens [29], yet such patients may also be likely to dropout. Consequently, we assessed the association between initial ART regimen and risk of dropout, adjusting for age, sex, year of ART initiation, and the CD4+ T cell count and hemoglobin concentrations at ART initiation, as the minimum sufficient adjustment set of confounders.

Between 14 and 41 % of patients were lacking data on at least one of the laboratory-measured predictors. As missing information could potentially bias our conclusions [30], we assessed whether missing data on any variable was associated with dropout. We then multiply imputed missing predictor values, using variables with complete information (age, sex, OI/comorbidity diagnoses, dropout status, and follow-up time), as well as the available data from the variables with missing values [31]. Categorical variables were imputed using multinomial logistic regression with the “augment” option to prevent perfect prediction [32]; numeric variables were imputed using predictive mean matching since they had skewed distributions [33, 34]; binary variables were imputed using logit models. Imputation procedures used iterative chained equations with 40 repetitions [35]. Analyses were performed in Stata 13 (Stata Corp, College Station, Texas, USA).

## Results

### Overall sample characteristics

Between January 2008 and December 2011, a total of 5,057 HIV-infected patients (64 % female) initiated ART. The median age was 33 years (IQR 28 to 40); 27.4 % had CD4+ T-cell counts <100 cells/μL (Table 1). Median follow-up was 24 months (IQR = 14 to 42, maximum follow-up = 64 months). Overall dropout was 26.9 % (established cumulative mortality = 2.3 %, loss to follow-up = 24.6 %), 5.6 % were transferred to other service providers, and 67.5 % were retained in care. The risk of dropout was not substantially different between those with data versus those without data on the variables that had missing data (Table 2).

**Table 1:** Characteristics of 5,057 patients who were analyzed in the study. The patients initiated ART in 2008 to 2011 at the Immune Suppression Syndrome Clinic in Mbarara, Uganda

Variable	
Age	33 (28–40)^a^
Sex, Female	63.9 %
Education	
No education	5.5 %
Primary	62.1 %
Secondary or more	32.5 %
Time to clinic	
≤1 h	14.8 %
1-2 h	20.4 %
2-3 h	21.9 %
≥4 h	43.0 %
Marital status	
Separated	13.2 %
Divorced	5.7 %
Widowed	16.8 %
Single	10.0 %
Married	54.4 %
Year of ART initiation	
2008	26.1 %
2009	19.6 %
2010	22.0 %
2011	32.2 %
Opportunistic infections	
Tuberculosis, history of	5.9 %
Kaposi’s sarcoma diagnosis	1.6 %
Cryptococcosis, history of	1.2 %
Esophagitis	2.2 %
Oral candidiasis	17.2 %
Chronic diarrhea	11.3 %
Weight loss ≥10 %	12.9 %
HIV-associated dementia	0.57 %
CD4+ T-cell counts (cells/μL)	
< 100	27.4 %
100–200	25.9 %
200–349	36.6 %
≥ 350	10.1 %
Hemoglobin (g/dl)	12.3 (11.0–13.7)
Neutrophils (x 10^3^ cells/μL)	1.7 (1.1–2.4)
Eosinophils (x 10^3^ cells/μL)	0.09 (0.04–0.20)
Platelets (x 10^3^ cells/μL)	208 (155–268)
Lymphocytes (x 10^3^ cells/μL)^b^	1.6 (1.1–2.0)
Initial ART regimens	
AZT, 3TC, NVP	58.5 %
AZT, 3TC, EFV	15.1 %
TDF, 3TC/FTC, EFV	19.4 %
TDF, 3TC/FTC, NVP	2.9 %
D4T, 3TC, EFV/NVP	4.1 %

*AZT* zidovudine, *3TC* lamivudine, *D4T* stavudine, *NVP* nevirapine, *EFV* efavirenz, *TDF* tenofovir

^a^Median (Interquartile range) unless otherwise specified

^b^Total lymphocyte count (TLC)

### Initial ART regimens

The majority of patients were initially treated with AZT-based regimens (for 58.5 % of the cohort, AZT was given alongside 3TC and NVP, and for 15.1 % AZT was given alongside 3TC and EFV). TDF-based regimens were initially given to 22.3 % overall (for 19.5 % TDF was combined with either 3TC or FTC and EFV and for 2.9 % NVP was used as the third drug). A small percent (4.1 %) received D4T-based regimens (with either 3TC and NVP or 3TC and EFV).

### Predictors of dropout from care

In the unadjusted analysis to evaluate predictors of dropout among those variables that are routinely collected from patients at ART initiation, we assessed 20 variables (shown in Table 1). Fifteen variables: sex, age, marital status, history of cryptococcosis, KS diagnosis, (recent) history of TB, weight loss ≥10 %, oral candidiasis, chronic diarrhea, HIV associated dementia, CD4+ T-cell count, hemoglobin concentration, platelet count, total lymphocyte count, and year of ART initiation predicted dropout at P <0.1. These were included in the multivariable adjusted proportional hazards regression model. Using a step-wise backward selection procedure, we removed variables from the model until 12 variables, all of which predicted dropout at P <0.05, remained (Table 3).

**Table 2:** Proportions with missing data and risk of dropout per variable for variables with missing data. The table shows the proportion of patients dropping out among those with data versus those without data in the full sample (N = 5,052) per variable for those variables which had some missing data

Variable	Missing data (%)	Dropout risk in those with data (%)	Dropout risk in those without data (%)
Education	31.1	18.8	17.2
Marital status	14.8	18.3	18.2
Time to clinic	29.3	19.4	15.5
CD4+ T cell count	21.2	17.8	20.0
Hemoglobin	28.2	18.3	18.3
Eosinophils	40.7	17.9	18.9
Neutrophils	33.5	18.3	18.2
Platelets	28.5	18.2	18.4
Total lymphocytes	32.5	18.3	18.2

**Table 3:** Hazard ratios of dropout per variables for variables that predicted dropout

	Unadjusted analysis		Adjusted analysis	
Variable	HR (95 % CI)	*P*	AHR^a^ (95 % CI)	*P*
Age				
16–28	1.3 (1.2–1.5)	<0.001	1.4 (1.2–1.6)	0.001
29–33	0.97 (0.83–1.1)	0.722	1.0 (0.685–1.2)	0.916
34–40	0.98 (0.84–1.1)	0.769	0.95 (0.80–1.1)	0.573
41–83	Ref.	Ref	Ref.	-
Sex				
Female	Ref.		Ref.	-
Male	1.2 (1.1–1.4)	<0.001	1.4 (1.2–1.6)	<0.001
Marital status				
Separated	1.4 (1.2–1.7)	<0.001	1.3 (1.1–1.5)	0.007
Divorced	1.4 (1.1–1.8)	0.005	1.3 (1.1–1.7)	0.017
Widowed	1.2 (0.90–1.3)	0.515	1.1 (0.88–1.3)	0.561
Single	1.6 (1.3–1.9)	<0.001	1.3 (1.1–1.6)	0.012
Married	Ref.	-	Ref.	-
Year of ART initiation				
2008	1.8 (1.5–2.0)	<0.001	1.6 (1.4–1.9)	<0.001
2009	1.6 (1.4–1.9)	<0.001	1.4 (1.2–1.7)	<0.001
2010	1.2 (1.0–1.5)	0.025	1.0 (0.82–1.2)	0.963
2011	Ref.	-	Ref.	-
Tuberculosis, history of	1.6 (1.4–2.0)	<0.001	1.4 (1.2–1.7)	<0.001
Kaposi’s sarcoma	4.3 (3.3–5.7)	<0.001	3.3 (2.5–4.5)	<0.001
Cryptococcosis, history of	2.4 (1.7–3.4)	<0.001	2.2 (1.4–3.3)	<0.001
Oral candidiasis	1.3 (1.1–1.5)	<0.001	-	-
Chronic diarrhea	1.3 (1.2–1.6)	<0.001	1.3 (1.1–1.5)	0.008
Weight loss ≥10 %	1.8 (1.6–2.1)	<0.001	1.5 (1.2–1.8)	<0.001
HIV-associated dementia	2.0 (1.2–12.8)	0.008	2.6 (1.5–4.6)	0.001
CD4+ T cell count				
<100 (cells/μL)	1.9 (1.5–2.4)	<0.001	-	-
100-199	1.2 (0.96–1.5)	0.107	-	-
200-349	1.0 (0.83–1.3)	0.768	-	-
≥350	Ref.	-	-	-
Hemoglobin concentration				
≤11.0 (g/dl)	2.0 (1.7–2.4)	<0.001	1.9 (1.6–2.2)	<0.001
11.1–12.4	1.4 (1.2–1.6)	<0.001	1.4 (1.1–1.6)	0.002
12.5–13.7	1.1 (0.91–1.3)	0.411	1.1 (0.94–1.4)	0.171
≥13.8	Ref.	-	Ref.	-
Platelet counts				
≤155 (cells/μL)	1.2 (1.0–1.4)	0.017	-	-
156–208	0.99 (0.84–1.2)	0.865	-	-
209–267	Ref.	-	-	-
268–720	1.1 (0.93–1.3)	0.301	-	-
Total lymphocyte count				
≤1.12 (x 10^3^ cells/μL)	1.6 (1.4–1.9)	<0.001	1.4 (1.2–1.7)	<0.001
1.13–1.6	1.3 (1.1–1.5)	0.003	1.2 (1.0–1.4)	0.048
1.61–2.0	Ref.	-	Ref.	-
≥2.01	1.3 (1.1–1.5)	0.001	1.1 (0.94–1.4)	0.171

*HR* hazard ratio, *AHR* adjusted hazard ratio, *CI* confidence interval

^a^For the variables that remained in the final adjusted model

A diagnosis of KS (HR = 3.3, 95 % CI 2.5 to 4.5); HIV-associated dementia (HR = 2.6, 95 % CI 1.5 to 4.6); history of cryptococcosis (HR = 2.2, 95 % CI 1.4 to 3.3); and reduced hemoglobin concentration (<11 g/dl versus ≥13.8 g/dl (HR = 1.9, 95 % CI 1.6 to 2.2) were strong predictors of dropout. Other independent predictors of dropout were: year of ART initiation; weight loss ≥10 %; reduced total lymphocyte count; chronic diarrhea; male sex; young age (≤28 years); and marital status (Table 3).

### Initial ART regimens and risk of dropout

In the unadjusted analysis, compared to those initially receiving AZT-based regimens, those receiving either TDF- or D4T-based regimens appeared to have a higher risk of dropout from treatment. After adjusting for age, sex, year of ART initiation, and the CD4+ T cell count and hemoglobin concentration at ART initiation, those initially receiving TDF-based regimens had a 1.4-fold higher risk of dropout (95 % CI 1.2 to 1.7) than those initially receiving AZT-based regimens. Those initially receiving D4T-based regimens had a 1.2-fold higher risk of dropout but the result was not statistically significant (95 % CI 0.92 to 1.5) (Table 4).

**Table 4:** Initial ART regimens and risk of dropout

	Unadjusted analysis		Adjusted analysis^b^	
Regimen backbone^a^	HR	*P*	AHR	*P*
AZT-based (reference)	-	-	-	-
TDF-based	1.2 (1.0–1.4)	0.022	1.4 (1.2–1.7)	<0.001
D4T-based^‡^	1.3 (0.98–1.6)	0.072	1.2 (0.92–1.5)	0.180

^a^AZT-based regimens contain zidovudine (AZT), lamivudine (3TC), and either nevirapine (NVP) or efavirenz (EFV); TDF-based regimens contain tenofovir (TDF), 3TC or FTC, and either NVP or EFV; D4T-based regimens contain stavudine (D4T), 3TC, and either NVP or EFV

^b^Adjusted for age, sex, year of ART initiation, and CD4+ T-cell count and hemoglobin concentration at ART initiation

## Discussion

In SSA, HIV-infected patients commonly drop out from treatment after initiating ART; the majority of dropouts are lost to follow-up [12]. This problem complicates the evaluation of HIV treatment services [36, 37], and interventions are needed to improve not only individual-level patient outcomes, but also outcome ascertainment. In this study, we assessed predictors of dropout from care among HIV-infected patients initiating ART at a public sector HIV treatment clinic in Uganda. Our data suggest that a number of variables that are routinely collected at ART initiation can predict dropout.

Correlates of death in HIV-infected patients such as cryptococcosis [38, 39] and a diagnosis of KS [40], as well as other proxies of HIV-disease progression at ART initiation, including reduced hemoglobin concentration and weight loss, [41] were especially predictive of dropout from care. As majority of dropouts in our analysis were lost to follow-up, we interpret these results to mean that many of the patients who got lost to follow-up are likely to have died.

This observation is consistent with observations from other programs and studies in the region. In a recent study in Ethiopia, predictors of attrition included severe immune deficiency at enrollment, a functional status of “bed-ridden” or “ambulatory”, compared to “working”, and male sex [42]. In another study in Zimbabwe, advanced HIV disease stage, and lower body weight at ART initiation also predicted attrition [43]. In a study which tracked a sample of those lost to follow-up, cumulative mortality among those lost to follow-up was 36 % at one year suggesting a high risk of death in those lost to follow-up [16].

Our observations would therefore suggest that earlier initiation of ART, before excessive HIV disease progression has occurred, may be one way to improve both individual-level patient outcomes, as well as outcome ascertainment in this setting [44]. We were unable to separately identify predictors of loss to follow-up from predictors of death (due to high rates of loss to follow-up in a context of inadequate ascertainment of death status). However, consistent with our observations, some previous studies suggest that young age, male sex, and marital status, as well as advanced HIV disease stage, predict loss to follow-up among ART initiators [24, 25].

Potentially, intensive follow-up for patients initiating ART may improve outcome ascertainment. For example, in research programs, where patients usually get intensively tracked, rates of loss to follow-up tend to be low (8 % at 3 years in one report) [45]. In contrast, in routine treatment programs, rates of loss to follow-up are high (e.g., 39 % at 3 years in a sample of all HIV-infected patients [16], and 30.8 % at 8 months in a sample of those co-infected with TB and HIV [17]). However, as intensively tracking all patients in a routine treatment program may be difficult, a better understanding of predictors of dropout could guide the development of predictive models that can be used to target interventions like intensive follow-up. In routine treatment programs, such models could be more useful if they predict both death and loss to follow-up as both are common negative outcomes in the context of ART in this setting.

Other interventions that can improve individual-level patient outcomes should also be investigated. For example, it may be that those dropping out require treatments over and above ART, which are not being provided in the present era. As some previous studies suggest that the immune reconstitution syndrome (IRIS) may lead to early death in some patients, especially those with cryptococcal disease [46] or KS [40], the role of IRIS and other risk factors for early death after ART initiation should be further investigated. This may aid the development of appropriate adjunctive interventions (i.e., other treatments over and above ART that might improve the survival of some HIV-infected ART initiators).

Other than the patients’ individual-level characteristics, some ART regimens also might conceivably increase risk of dropout. In a previous study which compared TDF-based regimens to AZT-based regimens, patients receiving a TDF-based regimen as initial therapy had an about 1.5-fold higher risk of death at or after 90 days from ART initiation, adjusting for age, sex, CD4+ cell count, HIV disease stage, body mass index, hemoglobin concentration, and serum creatinine at ART initiation [22]. In our data, we also found that patients on TDF-based regimens had a 1.4-fold higher risk of dropout than those on AZT-based regimens, although we were not able to adjust for any differences in baseline kidney function given that serum creatinine or other related data were not available. As a strength, our results were adjusted for all of age, sex, year of ART initiation, and CD4+ T cell count and hemoglobin concentration at ART initiation. In another previous study, D4T-based regimens were associated with a higher risk of symptomatic neuropathy than AZT-based regimens [28]. In our data, patients initially receiving D4T-based regimens had slightly increased risk of dropout, but their number was generally small and the result was not statistically significant; use of D4T in initial regimens at this clinic (and in Uganda generally [29]) has recently become rare.

A general limitation of our study is missing data. For example, if CBCs were not requested for patients that looked healthy; such patients may also be less likely to dropout, and the associations of CBC characteristics with dropout may be overestimated [30]. However, those with data were not substantially more likely to dropout than those without the data, and we used multiple imputation to impute values for the missing data. An additional limitation is that we were only able to assess a relatively small number of variables that were available in the clinic data; future studies assessing more variables are needed. Also, our findings are generally applicable to only routine treatment programs, where more complex variables that might be more predictive of outcomes tend to be unavailable.

## Conclusions

Among HIV infected patients initiating ART at a public sector clinic in SSA, several routinely collected variables predicted risk of dropout from treatment. Biological factors which usually predict death were especially predictive of dropout. As the majority of those dropping out in our study were lost to follow-up, our findings suggest that many of those getting lost to follow-up may have died. More studies are needed to identify appropriate interventions to improve individual-level patient outcomes, as well as outcome ascertainment, for HIV-infected patients initiating ART in this setting.

## Ethics approval and consent to participate

The study was approved by the Mbarara University of Science and Technology - Institutional Review Board (MUST-IRB). We used for this analysis data that had already been collected for clinical management, but the data were de-identified before being given to the analyst. Consequently, we applied to the local IRB for a waiver on the requirement for individual-level consent by patients to participate in this study.

## Consent for publication

As we used only de-identified data, we sought and were granted a waiver on the requirement for individual-level consent by patients regarding data publication. We sought for a waiver of individual-level consent because we felt that any attempts to individually contact patients would have been either more inconveniencing to the patients or in some cases difficult to implement.

## Data availability

The data that we used for this analysis can be accessed if requested from the corresponding author.

## Abbreviations

ART: antiretroviral therapy
AZT: zidovudine
CBC: complete blood count
D4T: stavudine
EFV: efavirenz
HR: hazard ratio
KS: kaposi’s sarcoma
NVP: nevirapine
SSA: Sub-Saharan Africa
TB: tuberculosis
3TC: lamivudine
TDF: tenofovir
TLC: total lymphocyte count

## References

[1] UNAIDS report on the global AIDS epidemic 2013 [http://www.unaids.org/sites/default/files/en/media/unaids/contentassets/documents/epidemiology/2013/gr2013/UNAIDS_Global_Report_2013_en.pdf]. Accessed 2/24/2015.

[2] THE HIV AND. AIDS UGANDA COUNTRY PROGRESS REPORT. 2014 [http://www.unaids.org/sites/default/files/country/documents/UGA_narrative_report_2015.pdf]. Accessed 12/22/2015.

[3] Epidemiological factsheets on HIV and sexually transmitted infections [http://data.unaids.org/publications/fact-sheets01/uganda_en.pdf]. Accessed 12/22/2015.

[4] Gregson S, Gonese E, Hallett TB, Taruberekera N, Hargrove JW, Lopman B (2010). HIV decline in Zimbabwe due to reductions in risky sex? Evidence from a comprehensive epidemiological review. Int J Epidemiol.

[5] Rehle TM, Hallett TB, Shisana O, Pillay-van Wyk V, Zuma K, Carrara H (2010). A decline in new HIV infections in South Africa: estimating HIV incidence from three national HIV surveys in 2002, 2005 and 2008. PLoS One.

[6] Murray CJ, Ortblad KF, Guinovart C, Lim SS, Wolock TM, Roberts DA (2014). Global, regional, and national incidence and mortality for HIV, tuberculosis, and malaria during 1990–2013: a systematic analysis for the Global Burden of Disease Study 2013. Lancet.

[7] UNAIDS: Access to Antiretroviral Therapy in Africa: Status Report on Progress Towards the 2015 Targets [http://www.unaids.org/sites/default/files/media_asset/20131219_AccessARTAfricaStatusReportProgresstowards2015Targets_en_0.pdf]. Accessed 2/24/2015.

[8] Boulle A, Schomaker M, May MT, Hogg RS, Shepherd BE, Monge S (2014). Mortality in patients with HIV-1 infection starting antiretroviral therapy in South Africa, Europe, or North America: a collaborative analysis of prospective studies. PLoS Med.

[9] Cohen MS, Chen YQ, McCauley M, Gamble T, Hosseinipour MC, Kumarasamy N (2011). Prevention of HIV-1 infection with early antiretroviral therapy. N Engl J Med.

[10] Rosen S, Fox MP, Gill CJ (2007). Patient retention in antiretroviral therapy programs in sub-Saharan Africa: a systematic review. PLoS Med.

[11] Koole O, Tsui S, Wabwire-Mangen F, Kwesigabo G, Menten J, Mulenga M (2014). Retention and risk factors for attrition among adults in antiretroviral treatment programmes in Tanzania, Uganda and Zambia. Trop Med Int Health.

[12] Fox MP, Rosen S (2010). Patient retention in antiretroviral therapy programs up to three years on treatment in sub-Saharan Africa, 2007–2009: systematic review. Trop Med Int Health.

[13] Estill J, Tweya H, Egger M, Wandeler G, Feldacker C, Johnson LF (2014). Tracing of patients lost to follow-up and HIV transmission: mathematical modeling study based on 2 large ART programs in Malawi. J Acquir Immune Defic Syndr.

[14] Henriques J, Pujades-Rodriguez M, McGuire M, Szumilin E, Iwaz J, Etard JF (2012). Comparison of methods to correct survival estimates and survival regression analysis on a large HIV African cohort. PLoS One.

[15] Egger M, Spycher BD, Sidle J, Weigel R, Geng EH, Fox MP (2011). Correcting mortality for loss to follow-up: a nomogram applied to antiretroviral treatment programmes in sub-Saharan Africa. PLoS Med.

[16] Geng EH, Glidden DV, Emenyonu N, Musinguzi N, Bwana MB, Neilands TB (2010). Tracking a sample of patients lost to follow-up has a major impact on understanding determinants of survival in HIV-infected patients on antiretroviral therapy in Africa. Trop Med Int Health.

[17] Nansera D, Bajunirwe F, Elyanu P, Asiimwe C, Amanyire G, Graziano FM (2012). Mortality and loss to follow-up among tuberculosis and HIV co-infected patients in rural southwestern Uganda. Int J Tuberc Lung Dis.

[18] Were MC, Shen C, Bwana M, Emenyonu N, Musinguzi N, Nkuyahaga F (2010). Creation and evaluation of EMR-based paper clinical summaries to support HIV-care in Uganda, Africa. Int J Med Inform.

[19] Koole O, Munthali L, Mhango B, Mpunga J, Glynn JR, Crampin AC (2014). Impact of changing diagnostic criteria for smear-positive tuberculosis: a cohort study in Malawi. Int J Tuberc Lung Dis.

[20] Laker-Oketta MO, Wenger M, Semeere A, Castelnuovo B, Kambugu A, Lukande R, Asirwa FC, Busakhala N, Buziba N, Diero L et al.: Task Shifting and Skin Punch for the Histologic Diagnosis of Kaposi's Sarcoma in Sub-Saharan Africa: A Public Health Solution to a Public Health Problem. Oncology. 2015;89(1):60-5. 10.1159/000375165 PMC4485601 25765812

[21] Longley N, Muzoora C, Taseera K, Mwesigye J, Rwebembera J, Chakera A (2008). Dose response effect of high-dose fluconazole for HIV-associated cryptococcal meningitis in southwestern Uganda. Clin Infect Dis.

[22] Chi BH, Mwango A, Giganti MJ, Sikazwe I, Moyo C, Schuttner L (2011). Comparative outcomes of tenofovir-based and zidovudine-based antiretroviral therapy regimens in Lusaka, Zambia. J Acquir Immune Defic Syndr.

[23] Geng EH, Emenyonu N, Bwana MB, Glidden DV, Martin JN (2008). Sampling-based approach to determining outcomes of patients lost to follow-up in antiretroviral therapy scale-up programs in Africa. Jama.

[24] Meloni ST, Chang C, Chaplin B, Rawizza H, Jolayemi O, Banigbe B (2014). Time-Dependent Predictors of Loss to Follow-Up in a Large HIV Treatment Cohort in Nigeria. Open Forum Infectious Diseases.

[25] Berheto TM, Haile DB, Mohammed S (2014). Predictors of Loss to follow-up in Patients Living with HIV/AIDS after Initiation of Antiretroviral Therapy. North Am J Med Sci.

[26] Ochieng-Ooko V, Ochieng D, Sidle JE, Holdsworth M, Wools-Kaloustian K, Siika AM (2010). Influence of gender on loss to follow-up in a large HIV treatment programme in western Kenya. Bull World Health Organ.

[27] Geng EH, Glidden DV, Bangsberg DR, Bwana MB, Musinguzi N, Nash D (2012). A causal framework for understanding the effect of losses to follow-up on epidemiologic analyses in clinic-based cohorts: the case of HIV-infected patients on antiretroviral therapy in Africa. Am J Epidemiol.

[28] McGrath CJ, Njoroge J, John-Stewart GC, Kohler PK, Benki-Nugent SF, Thiga JW (2012). Increased incidence of symptomatic peripheral neuropathy among adults receiving stavudine- versus zidovudine-based antiretroviral regimens in Kenya. J Neurovirol.

[29] Duber HC, Dansereau E, Masters SH, Achan J, Burstein R, DeCenso B (2015). Uptake of WHO recommendations for first-line antiretroviral therapy in Kenya, Uganda, and Zambia. PLoS One.

[30] Oksuzyan A, Petersen I, Stovring H, Bingley P, Vaupel JW, Christensen K (2009). The male–female health-survival paradox: a survey and register study of the impact of sex-specific selection and information bias. Ann Epidemiol.

[31] Royston P (2004). Multiple imputation of missing values. Stata J.

[32] Royston P (2009). Multiple imputation of missing values: further update of ice, with an emphasis on categorical variables. Stata J.

[33] Morris TP, White IR, Royston P (2014). Tuning multiple imputation by predictive mean matching and local residual draws. BMC Med Res Methodol.

[34] Gerko V, Frank LE, Jeroen P, Stef VB (2014). Predictive mean matching imputation of semicontinuous variables. Statistica Neerlandica.

[35] Graham JW, Olchowski AE, Gilreath TD (2007). How many imputations are really needed? Some practical clarifications of multiple imputation theory. Prev Sci.

[36] Verguet S, Lim SS, Murray CJ, Gakidou E, Salomon JA (2013). Incorporating loss to follow-up in estimates of survival among HIV-infected individuals in sub-Saharan Africa enrolled in antiretroviral therapy programs. J Infect Dis.

[37] Schomaker M, Gsponer T, Estill J, Fox M, Boulle A (2014). Non-ignorable loss to follow-up: correcting mortality estimates based on additional outcome ascertainment. Stat Med.

[38] Lewden C, Drabo YJ, Zannou DM, Maiga MY, Minta DK, Sow PS (2014). Disease patterns and causes of death of hospitalized HIV-positive adults in West Africa: a multicountry survey in the antiretroviral treatment era. J Int AIDS Soc.

[39] Adeyemi B, Ross A (2014). Profile and mortality outcome of patients admitted with cryptococcal meningitis to an urban district hospital in KwaZulu-Natal, South Africa. J Int AIDS Soc.

[40] Letang E, Lewis JJ, Bower M, Mosam A, Borok M, Campbell TB (2013). Immune reconstitution inflammatory syndrome associated with Kaposi sarcoma: higher incidence and mortality in Africa than in the UK. Aids.

[41] Erikstrup C, Kallestrup P, Zinyama R, Gomo E, Mudenge B, Gerstoft J (2007). Predictors of mortality in a cohort of HIV-1-infected adults in rural Africa. J Acquir Immune Defic Syndr.

[42] Mekuria LA, Prins JM, Yalew AW, Sprangers MA, Nieuwkerk PT (2015). Retention in HIV Care and Predictors of Attrition from Care among HIV-Infected Adults Receiving Combination Anti-Retroviral Therapy in Addis Ababa. PLoS One.

[43] Mutasa-Apollo T, Shiraishi RW, Takarinda KC, Dzangare J, Mugurungi O, Murungu J (2014). Patient retention, clinical outcomes and attrition-associated factors of HIV-infected patients enrolled in Zimbabwe’s National Antiretroviral Therapy Programme, 2007–2010. PLoS One.

[44] Lahuerta M, Ue F, Hoffman S, Elul B, Kulkarni SG, Wu Y (2013). The problem of late ART initiation in Sub-Saharan Africa: a transient aspect of scale-up or a long-term phenomenon?. J Health Care Poor Underserved.

[45] Hunt PW, Cao HL, Muzoora C, Ssewanyana I, Bennett J, Emenyonu N (2011). Impact of CD8+ T-cell activation on CD4+ T-cell recovery and mortality in HIV-infected Ugandans initiating antiretroviral therapy. Aids.

[46] Boulware DR, Meya DB, Muzoora C, Rolfes MA, Huppler Hullsiek K, Musubire A (2014). Timing of antiretroviral therapy after diagnosis of cryptococcal meningitis. N Engl J Med.

